# Long non-coding RNA (lncRNA) PGM5P4-AS1 inhibits lung cancer progression by up-regulating leucine zipper tumor suppressor (LZTS3) through sponging microRNA miR-1275

**DOI:** 10.1080/21655979.2020.1860492

**Published:** 2020-12-31

**Authors:** Junpeng Feng, Jianhang Li, Peng Qie, Zhenhua Li, Yanzhao Xu, Ziqiang Tian

**Affiliations:** a Department of Thoracic Surgery, The Fourth Hospital of Hebei Medical University, Shijiazhuang, P.R. China; b Department of Thoracic Surgery, Hebei Chest Hospital, Shijiazhuang, P.R. China; c Department of Thoracic Surgery, Hebei General Hospital, Shijiazhuang, P.R. China

**Keywords:** Lung cancer, long non-coding RNA PGM5P4-AS1, microRNA miR-1275/leucine zipper putative tumor suppressor 3, proliferation, metastasis

## Abstract

It is necessary to explore new molecules for the improvement of precise diagnosis and antitumor therapies in lung cancer. LncRNAs (long non-coding RNAs) play an important role in the regulation of cancer cell malignant behavior and tumor development. In this work, we found that a newly discovered lncRNA, lncRNA PGM5P4-AS1, was lower expressed in lung cancer tissues than adjacent tissues. Then, the lncRNA PGM5P4-AS1 was overexpressed or knocked-down in different lung cancer cells, and its effects on the malignant phenotypes were measured by 3-(4, 5-Dimethylthiazol-2-yl)-2, 5-diphenyltetrazolium bromide (MTT) assay, cell cycle assay, wound healing assay, and transwell assay. The results showed that the overexpression of PGM5P4-AS1 inhibited lung cancer cell proliferation, migration, and invasion activities, while these abilities were prominently promoted by the interference of PGM5P4-AS1. Further, the growth of lung cancer tumors in nude mice was also inhibited by PGM5P4-AS1 overexpression. In mechanism, PGM5P4-AS1 has the binding site of miR-1275 and could positively regulate the expression of LZTS3 via sponging miR-1275. In conclusion, PGM5P4-AS1 could be a potential precise diagnosis and therapeutic target biomarker of lung cancer.

## GRAPHICAL ABSTRACT

**Figure:**
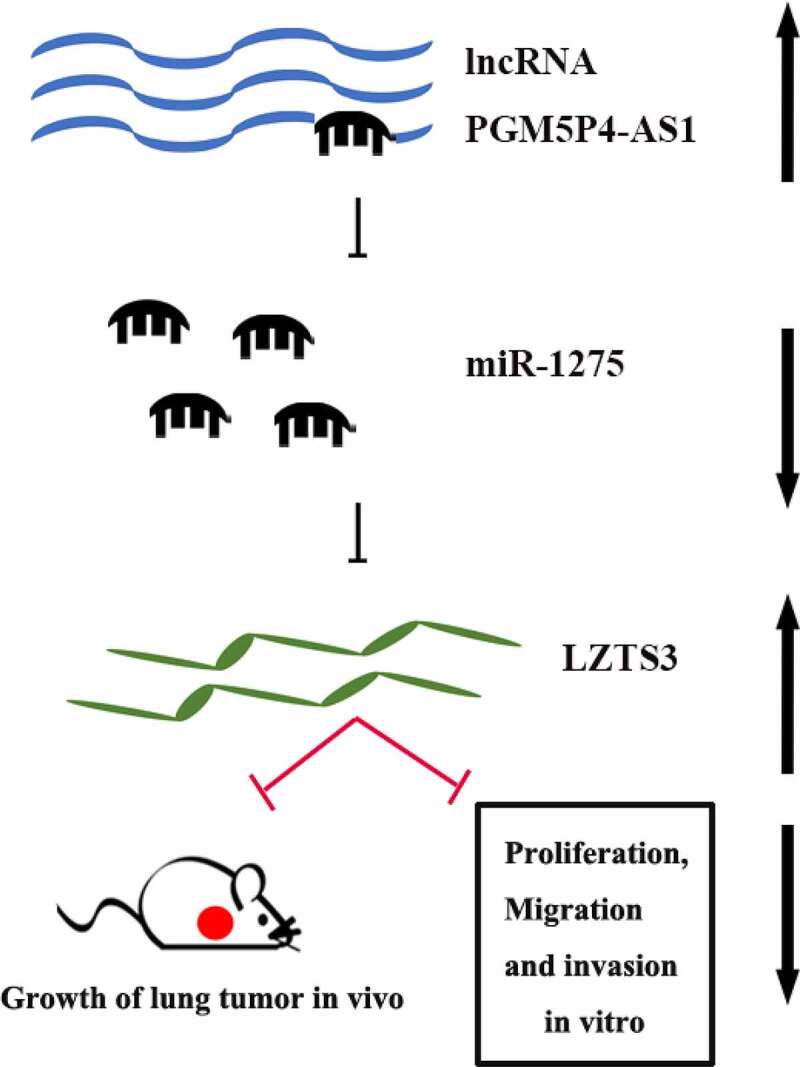

## Introduction

Lung cancer is considered the most cause of malignant tumor-related death worldwide with a high level of mortality for 18.4% [1]. Lung cancer can be divided into small cell lung carcinoma (SCLC) and non-small cell lung carcinoma (NSCLC), which is further divided into adenocarcinoma (38.5% of all lung carcinoma), large cell carcinoma (2.9% of all lung cancer cases), and squamous cell carcinoma (20% of all lung cancer cases) [2]. Surgery, chemotherapy, and medication are the major therapeutic strategies to treat lung cancer. Many patients relapse and succumb within a short time because of poor treatments and advanced stages of diagnosis [3]. Molecular heterogeneity in lung cancer is one of the reasons for these situations [4,5]. The dysregulated expression of cancer markers has been strongly linked to oncogenes or anti-tumor genes, that participate in carcinogenesis [6,7]. Therefore, it is necessary to extend the potential cancer-associated molecule library to improve the precise diagnosis and antitumor therapies in lung cancer.

Long non-coding RNAs (lncRNAs) are non-coding ribonucleotides that longer than 200 nucleotides. Resembled mRNA, some LncRNAs have 5ʹ-7 m7GPPPN cap and polt A tail, but they still have no function to encode proteins [8–10]. Most lncRNAs are active in the nucleus instead of the cytoplasm, and it is logical for lncRNAs to act a pivotal part in biochemical processes and genome activities [11]. LncRNAs can competitively combine with miRNAs as competing endogenous RNAs (ceRNAs) [12]. Studies have proven that many lncRNAs act as tumor suppressors and biomarkers for precise diagnosis, as well as regulate the tumor progression in various cancers [13–15]. Zhou et al. [16] found that lncRNA PANDAR had high expression levels in lung cancer, colorectal cancer, renal cell carcinoma, and so forth. The overexpression of PANDAR reduced the growth of tumors and affected mice survival rates and tumor cellular processes.

LncRNA PGM5P4-AS1 is a lncRNA with a length of 950 bp and might be an anti-cancer biomarker in many cancers. For example, Zhang et al. [17] reported that PGM5P4-AS1 was associated with diagnosis and prognosis in hepatocellular carcinoma by bioinformatics analysis. Zhang et al. [18] found that PGM5P4-AS1 might be a potential lncRNA biomarker via the construction of a ceRNAs network in rectal adenocarcinoma. Ouyang et al. [19] demonstrated that PGM5P4-AS1 was down-regulated in breast cancer by bioinformatics analysis and might be related to chemo-resistance. However, most of these studies on PGM5P4-AS1 in various cancers are performed by bioinformatics analysis and are rarely verified by experiments. In particular, the effects of PGM5P4-AS1 in lung cancer has not been reported. Through bioinformatics analysis, we found that PGM5P4-AS1 is significantly down-regulated in lung adenocarcinoma and lung squamous cell carcinoma; thus, we speculated that PGM5P4-AS1 might play a role in the biological functions of lung cancer.

In the current study, we aimed to explore the effects of PGM5P4-AS1 on lung cancer progression. We found that PGM5P4-AS1 was low expressed in lung cancer tissues by quantitative real-time PCR and then we artificially regulated the expression of PGM5P4-AS1. The modulation of PGM5P4-AS1 influenced the growth of lung cancer xenograft tumors *in vivo* as well as cell proliferation, migration, and invasion *in vitro* (measured by 3-(4,5-dimethylthiazol-2-yl)-2,5-diphenyltetrazolium bromide (MTT) assay, cell cycle arrest, wound healing, and transwell assay). Further luciferase reporter assay suggested that these effects were related to its regulation on the miR-1275/LZTS3 axis. The evidence revealed that PGM5P4-AS1 could act as a potential therapeutic target biomarker for lung cancer.

## Materials and methods

### Tissues and cell lines

This research was approved by the Ethics Committee of The Fourth Hospital of Hebei Medical University. Five pairs of lung cancer tissues and adjacent tissues were derived from patients in the Fourth Hospital of Hebei Medical University with signed informed consent. The tissue samples were preserved at −80°C until subsequent detection. Lung cell lines A549, SK-MES-1, NCI-H1437, and NCI-H1975 were purchased from Procell Life Science&Technology Co., Ltd (Wuhan, China), and NCI-H2170 was purchased from Zhongqiaoxinzhou Biotech Co., Ltd (Shanghai, China). A549 was cultured in Ham’s F-12 K (PM150910, Procell Life Science) with 10% fetal bovine serum (FBS; SH30084.03, Hyclone, USA), while SK-MES-1 was maintained in MEM (41,500–067, Gibco, USA) with 10% FBS. NCI-H1437, NCI-H2170, and NCI-H1975 were grown in RPMI1640 (31,800–014, Gibco) with 10% FBS. All cells were cultured in an incubator (HF-90, Shanghai Lishen, China) of 95% humidity, 5% CO_2_ at 37°C.

### RNA interference, vector construction, and transfection

Small interfering RNA (siRNA) was carried out for the silence of PGM5P4-AS1, which were synthesized by GenScript Co., Ltd (Nanjing, China). siRNA sequences are presented in Table 1. The overexpression vectors pcDNA3.1 (V79520, ThermoFisher Scientific, USA) of PGM5P4-AS1 were constructed. Cells were seeded into 6-well plates. 50 pmol DNA, 1 μg vectors, and 6 μL Lipofectamine 2000 Transfection Reagent (11,668–019, Invitrogen, USA) were added to cells in each well at 37°C for 20 min.

**Table 1.** Synthetic siRNA sequences

siRNAs	Sequences (5'-3')
PGM5P4-siRNA1 (F)	GGCCAGCAUAAGAAGGUGATT
PGM5P4- siRNA1 (R)	UCACCUUCUUAUGCUGGCCTT
PGM5P4-siRNA2 (F)	GGGAGAGUAUCCAGAAACATT
PGM5P4-siRNA2 (R)	UGUUUCUGGAUACUCUCCCTT
PGM5P4-siRNA3 (F)	GAGUUCCUAAUGACAGACATT
PGM5P4-siRNA3 (R)	UGUCUGUCAUUAGGAACUCTT
Negative control (F)	UUCUCCGAACGUGUCACGUTT
Negative control (R)	ACGUGACACGUUCGGAGAATT

Note: F, forward; R, reverse.

### Quantitative real-time PCR (qPCR)

Total RNA was extracted by the Total RNA extraction kit (DP419, Tiangen Biotech, China), then the RNA was reversed-transcribed into cDNA with super M-MLV reverse transcriptase (NG212, Tiangen Biotech) . Amplification was performed by 2× Taq PCR MasterMix (KT201, Tiangen Biotech) at the presence of SYBR Green (SY1020, Solarbio, China) as per the users’ instructions. The above procedures were set for triplicate for each system. Primers were synthesized by GenScript Co., Ltd, and primer sequences are shown in Table 2. The PCR reaction was performed on Exicycler TM 96 (Bioneer, Korea).

**Table 2.** qPCR primer sequences

Gene	Primer sequences (5'-3')
PGM5P4-AS1 (F)	GGGGCAGAGGGACTATGTT
PGM5P4-AS1 (R)	GAGGCTGAGGTGGGAGGAT
LZTS3 (F)	CACCGCAGTATCGTGAGCC
LZTS3 (R)	GGAAATTCTTGGGTACGACAGG
GAPDH (F)	GACCTGACCTGCCGTCTAG
GAPDH (R)	AGGAGTGGGTGTCGCTGT
hsa-miR-1275 (F)	GAACCTGGTAGTGGGGGAGA
hsa-miR-1275 (R)	GTGCAGGGTCCGAGGTATTC
U6 (F)	GCTTCGGCAGCACATATACT
U6 (R)	GTGCAGGGTCCGAGGTATTC

Note: F, forward; R, reverse.

PGM5P4-AS1 inhibited the growth of tumors in nude mice and suppressed the abilities of cell proliferation, migration as well as invasion of lung cancer cells. In mechanism, PGM5P4-AS1 performed antitumor function by up-regulating the expression of LZTS3 via sponging miR-1275.

### 3-(4,5-Dimethylthiazol-2-yl)-2,5-diphenyltetrazolium bromide (MTT) assay

3 × 10^3^ cells were seeded in a 96-well plate for each well. There were five repeats for each system. Cells were cultured in a completed medium with 0.5 mg/mL MTT (C0009, Beyotime, China) for 4.5 h after being transfected for 0 h, 24 h, 48 h, 72 h, and 96 h. Then, 150 μL DMSO (D-5879, Sigma, USA) was added to the 96-well plate for each well and incubated for 10 min. The absorbance at 570 nm was measured by a Microplate spectrophotometer (ELx-800, Biotek Instruments, USA).

### Cell cycle arrest

The serum-starved A549 and SK-MES-1 cells were washed with phosphate buffer saline (PBS) after being transfected for 48 h. Then, cells were fixed with 70% cold ethanol and kept at 4°C for 2 h. PBS was used to wash cells again and resuspend cells. Cells were stained with 25 μL propidium iodide (PI; C1052-2, Beyotime) and incubated with 10 μL RNase (C1052-3, Beyotime) at 37°C for 30 min. The cell cycle was detected by the flow cytometer (NovoCyte, ACEA Biosciences, USA).

### Xenograft model in nude mice and vector treatment

Male BALB/c nude mice were purchased from Beijing HFK Bioscience Co., Ltd (Beijing, China). Mice feeding and animal experiments followed The Guideline for the Care and Use of Laboratory Animals. SK-MES-1 cells were collected and washed with PBS twice and resuspended with a completed medium. 2 × 10^6^ cells were subcutaneously injected into the right armpit of nude mice. PGM5P4-AS1-OE vectors or control vectors (50 μg per mouse) were injected into mice tail vein three times a week after tumors reached the volume of 60–90 mm^3^. Six mice were set for each treatment.

### Immunohistochemistry

Tumor sections were incubated with goat serum (SL038, Solarbio) at room temperature for 15 min. Ki67 antibodies (diluted 1:100; A2094, ABclonal, China) were added overnight to the top of the section. The tumor sections we washed twice by PBS and incubated with a goat anti-rabbit IgG (H + L) antibody (diluted 1:500; #31,460, ThermoFisher Scientific) at 37°C for an hour. Then, the DAB Substrate kit (DA1010, Solarbio) was used for color rendering, and HE Staining Kit (G1120, Solarbio) was used to stain again. The positive staining of ki67 was photographed by a microscope (BX53, Olympus, Japan).

### Wound healing assay

After 48 h of transfection, cells were cultured with serum-free medium. Equal numbers of cells were planted in cell-cultured plates. Two hundred microliter pipette tips were used to draw a horizontal line when there were 80–90% of cells covered the bottom of the cultured plates. The cells were washed by a serum-free medium to remove the exfoliated cells. Then, the relative position of cells was photographed by a microscope (BX53, Olympus) at 0 h and 24 h, respectively.

### Transwell assay

A549 and SK-MES-1 cells were collected and resuspended with serum-free medium after being transfected for 48 h. 2.5 × 10^4^ cells were seeded into upper chambers of transwell inserts (3422, Corning, USA). There were 800 μL 10% FBS of the medium in the lower chambers for each well. Cells in transwell chambers were incubated for 24 h and stained with 0.5% crystal violet (0528, Amresco, USA). There were three repeats for each treatment. After that, cells in the upper chambers were wiped off, and the cell number of lower chambers were counted and photographed by a microscope (BX53, Olympus)

### Western blot

Total protein was extracted from A549 and SK-MES-1 cells after being transfected for 48 h. Protein concentration was detected by a BCA protein assay kit (PC0020, Solarbio). 10–20 μg protein was fractionated by SDS-PAGE electrophoreses. Then, the bands were transferred to polyvinylidene fluoride (PVDF) membranes (IPVH00010, Millipore, USA). After being blocked with 5% nonfat milk, protein blots were incubated with primary antibodies at 4°C overnight. The primary antibodies were cyclinD1 (diluted 1:1000; #55,506, CST, USA), MMP-2 (diluted 1:500; 10,373-2-AP, Proteintech, China), LZTS3 (diluted 1:1000; DF7557, Affinity Biosciences, China), Bcl-2 (diluted 1:1000, #3498, CST), Bcl-xl (diluted 1:1000; #24,780, CST) and GAPDH (diluted 1:10,000; 60,004-1-Ig, Proteintech). Then, the specific bands were further incubated with secondary antibodies IgG-HRP (diluted 1:3000; SE131, SE134, Solarbio) at room temperature for 45 minutes. The target protein expression was analyzed by Gel-Pro-Analyzer (WD-9413B, Beijing Liuyi Biotechnology, China).

### Luciferase reporter assay

We constructed the wild-type PGM5P4-AS1 vectors (wt-PGM5P4-AS1) that contained the binding sites of miR-1275 and the mutant PGM5P4-AS1 vectors (mut-PGM5P4-AS1) with binding site mutations. Wt-PGM5P4-AS1 and mut-PGM5P4-AS1 were transfected to the same 293 T cells (Zhongqiaoxinzhou Biotech) with NC mimic or miR-1275 mimic, respectively. Four groups were for the assay as followed: wt-PGM5P4-AS1+ NC mimic, mut-PGM5P4-AS1+ NC mimic, wt-PGM5P4-AS1+ miR-1275 mimic and mut-PGM5P4-AS1+ miR-1275 mimic. After that, cells were collected at 48 h after transfection. The ratio of firefly to renilla fluorescence intensity was detected as the relative luciferase intensity by the Dual-Luciferase Reporter Assay System (E1910, Promega Corporation, USA).

### Statistics analysis

Statistics were performed by using GraphPad Prism 8.0. Data were presented as mean ± standard deviation. A comparison of two treatments was analyzed with two-side t-tests, and multiple treatments were analyzed with one-way ANOVA. p < 0.05 was suggested statistically significant.

## Result

### The expression of PGM5P4-AS1 was increased in lung cancer tissues

**Figure 1.**
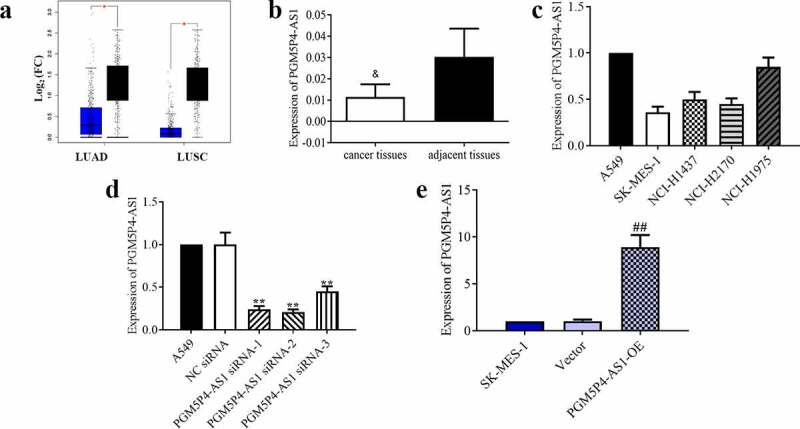
**The expression of PGM5P4-AS1 was increased in lung cancer tissues**. (a), Boxplot of PGM5P4-AS1 in LUAD and LUSC; LUAD, lung adenocarcinoma; LUSC, lung squamous cell carcinoma; FC, fold change (*p < 0.05 *vs*. normal tissues). Blue: tumor tissue; black: normal tissue. (b-c), Relative expression levels of PGM5P4-AS1 were measured by qRT-PCR assay in lung cancer tissues and adjacent tissues, as well as lung cancer cell lines, respectively (&p < 0.05 *vs*. adjacent tissues). (d-e), Transfection efficiency was detected after A549 and SK-MES-1 cells were transfected with PGM5P4-AS1 siRNA or PGM5P4-AS1-OE for 48 h, respectively (**p < 0.01 *vs*. NC siRNA, ##p < 0.01 *vs*. vector)

The bioinformatics analysis showed that PGM5P4-AS1 was lower expressed in lung adenocarcinoma (LUAD) and lung squamous cell carcinoma (LUSC) tumor tissues than in normal tissues (p < 0.05; Figure 1(a)). Our qPCR results further confirmed that PGM5P4-AS1 was significantly down-regulated in lung cancer tissues compared to adjacent tissues (0.01±0.01 *vs*. 0.03±0.01; p < 0.05; Figure 1(b)). We then detected the relative expression levels of PGM5P4-AS1 in several lung cancer cell lines by qPCR. As shown in Figure 1(c), PGM5P4-AS1 was highly expressed in A549 cells and low expressed in SK-MES-1 cells (1.00±0.00 *vs*. 0.36±0.06). Thus, PGM5P4-AS1 was knocked-down in A549 cells and up-regulated in SK-MES-1 cells, then the expression of PGM5P4-AS1 was detected by qPCR. The results showed that the relative expression levels of PGM5P4-AS1 were significantly down-regulated in A549 cells by siRNA transfection (0.24±0.04, 0.21±0.03, 0.45±0.06 *vs*. 1.00±0.14; p < 0.01; Figure 1(d)), and it was significantly up-regulated in SK-MES-1 cells by transfection with the PGM5P4-AS1 overexpression vectors (8.94±1.28 *vs*. 1.04±0.17; p < 0.01; Figure 1(e)).

### PGM5P4-AS1 suppressed the proliferation of lung cancer cells

**Figure 2.**
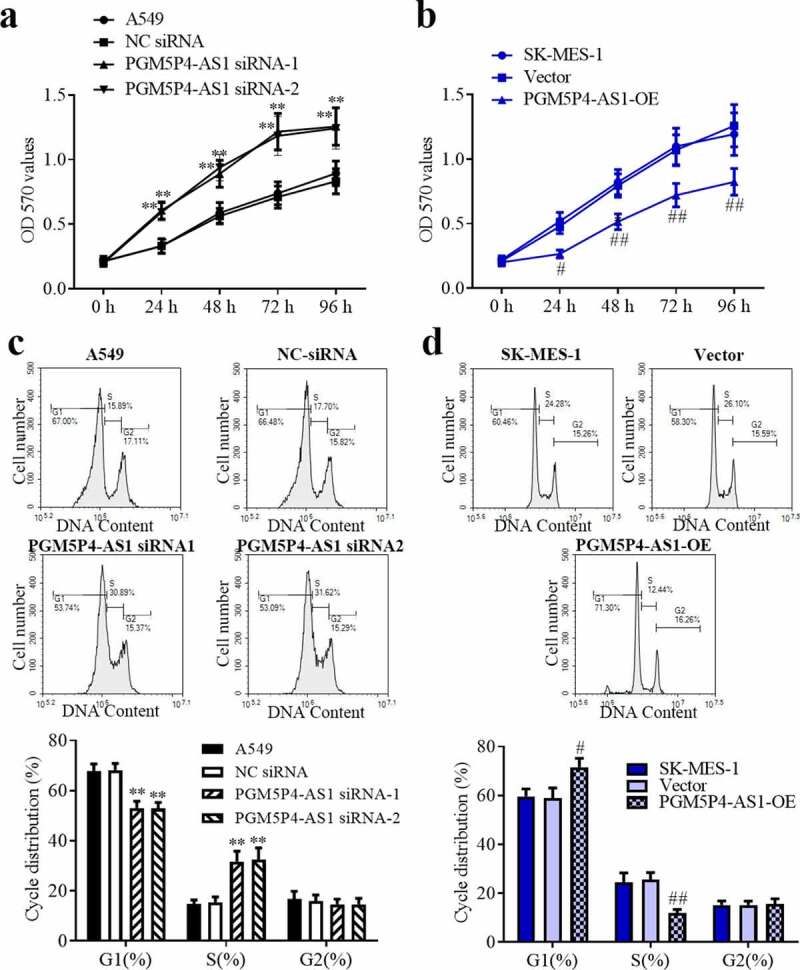
**PGM5P4-AS1 suppressed the proliferation of lung cancer cells. (**a-b), Cell proliferation of A549 and SK-MES-1 cells was measured by MTT assay after being transfected with PGM5P4-AS1 siRNA or PGM5P4-AS1-OE for 0 h, 24 h, 48 h, 72 h, or 96 h (#p < 0.05 *vs*. vector, ##p < 0.01 *vs*. vector, **p<0.01 *vs*. NC siRNA). (c-d), Cell cycles arrest of A549 and SK-MES-1 cells was measured by flow cytometer after being transfected with PGM5P4-AS1 siRNA or PGM5P4-AS1-OE for 48 h (#p < 0.05 *vs*. vector, ##p<0.01 *vs*. vector, **p<0.01 *vs*. NC siRNA)

The proliferation ability of the lung cancer cells was measured by MTT assay. As shown in Figure 2(a), from 24 to 96 h, the results of the MTT assay demonstrated that the interference in PGM5P4-AS1 expression in A549 cells significantly promoted cell proliferation (p < 0.01). And Figure 2(b) revealed that PGM5P4-AS1 overexpression significantly inhibited the proliferation of SK-MES-1 cells (from 24 to 96 h; p < 0.01). To determine whether the effect caused by PGM5P4-AS1 was associated with the cell cycle, flow cytometry was performed. The results indicated that the knock-down of PGM5P4-AS1 decreased G1 phase proportion (53.11%±2.66%, 52.90%±2.41% *vs*. 68.19%±2.61%) and increased S phase proportion (31.73%±4.11%, 32.41%±4.75% *vs*. 15.47%±2.11%; Figure 2(c)). Meanwhile, the overexpression of PGM5P4-AS1 increased the G1 phase proportion (71.63%±3.61% *vs*. 58.97%±4.14%) and reduced the S phase proportion (11.88%±1.48% *vs*. 25.6%±2.88%; Figure 2(d)). These results demonstrated that PGM5P4-AS1 can repress the proliferation of lung cancer cells.

### *PGM5P4-AS1 inhibited the* in vivo *growth of lung cancer*

**Figure 3.**
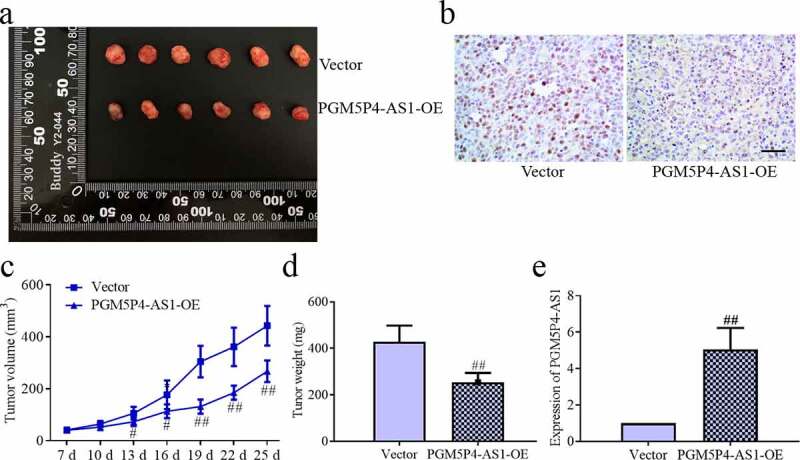
**PGM5P4-AS1 inhibited the *in vivo* growth of lung cancer. (**a), PGM5P4-AS1-OE or untreated vectors were injected in nude mice with xenograft tumors. (b), Cell proliferation ability of tumor was evaluated via ki67 immunohistochemistry (×400). The scale bar represented 50 μm. (c), Volume of the excised tumor (#p < 0.05 *vs*. vector, ##p < 0.01 *vs*. vector). (d), Weight of the excised tumor (##p < 0.01 *vs*. vector). (e), Relative expression of PGM5P4-AS1 by qRT-PCR in the tumor (##p < 0.01 *vs*. vector)

The nude mice xenograft tumors assay was employed to evaluate the effects of PGM5P4-AS1 on cancer growth *in vivo*. The results displayed that the over-expression of PGM5P4-AS1-OE significantly suppressed the growth of the tumor, as evidenced by the reduced tumor volume from 13th to 25th and tumor weight (254.17 mg±39.85 mg *vs*. 428.50 mg±69.34 mg; p < 0.01, Figure 3(a, c and d)). The expression of ki67 was detected to evaluate the cell proliferation activity of the tumors. Figure 3(b) reveals that the expression of ki67 was reduced by the up-regulation of PGM5P4-AS1. And as shown in Figure 3(e), the qPCR results verified the overexpression of PGM5P4-AS1 in SK-MES-1 xenograft tumors (5.06±1.18 *vs*. 1.00±0.00; p < 0.01).

### PGM5P4-AS1 inhibited the migration and invasion activities of lung cancer cells

**Figure 4.**
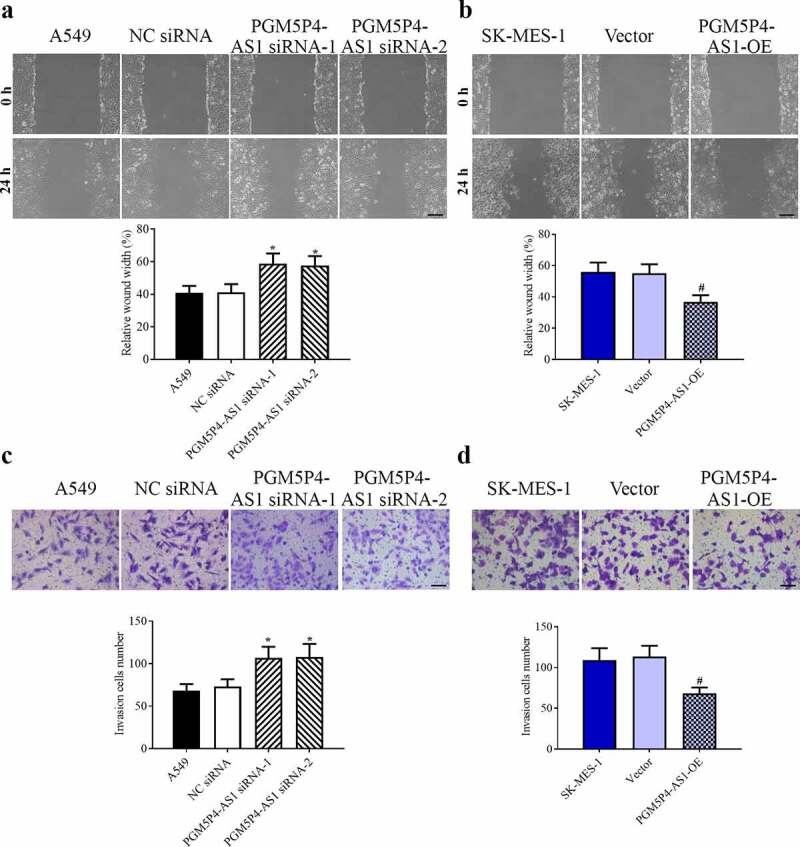
**PGM5P4-AS1 inhibited the migration and invasion activities of lung cancer cells. (**a), The migration ability of A549 cells was assessed by wound healing assay, and the results showed that interference in the expression of PGM5P4-AS1 could promote cell migration (×100, *p < 0.05 *vs*. NC siRNA). The scale bar represented 200 μm. (b), The results of the migration ability of SK-MES-1 cells showed that the overexpression of PGM5P4-AS1 could inhibit cell migration (×100, #p < 0.05 *vs*. vector). The scale bar represented 200 μm. (c), Invasion abilities were measured by transwell assay, the results showed that interference in the expression of PGM5P4-AS1 could promote cell invasion (×200, *p < 0.05 *vs*. NC siRNA). The scale bar represented 100 μm. (d), The overexpression of PGM5P4-AS1 could inhibit the cell invasion (×200, #p < 0.05 *vs*. vector). The scale bar represented 100 μm

As shown in Figure 4(a), the wound healing results showed that the knocked-down of PGM5P4-AS1 significantly enhanced the migration activity of A549 cells (relative wound width: 58.75%±6.30%, 57.63%±5.81% *vs*. 41.24%±5.02%; p < 0.05). And Figure 4(b) revealed that the overexpression of PGM5P4-AS1 significantly inhibited cell migration ability in SK-MES-1 cells (relative wound width: 36.93%±4.20% *vs*. 55.05%±5.78%; p < 0.05). According to the results of the transwell assay, we found that the invasive activity of the lung cancer cells was increased by the down-regulation of PGM5P4-AS1 (the invasion cell number: 106.80±12.96, 107.73±15.50 *vs*. 73.00±8.59) and decreased by the up-regulation of PGM5P4-AS1 (the invasion cell number: 68.07±7.37 *vs*. 113.27±13.31;). These results suggested that PGM5P4-AS1 suppressed the migration and invasion of lung cancer cells.

### PGM5P4-AS1 decreased the miR-1275 level and promoted the expression of LZTS3

**Figure 5.**
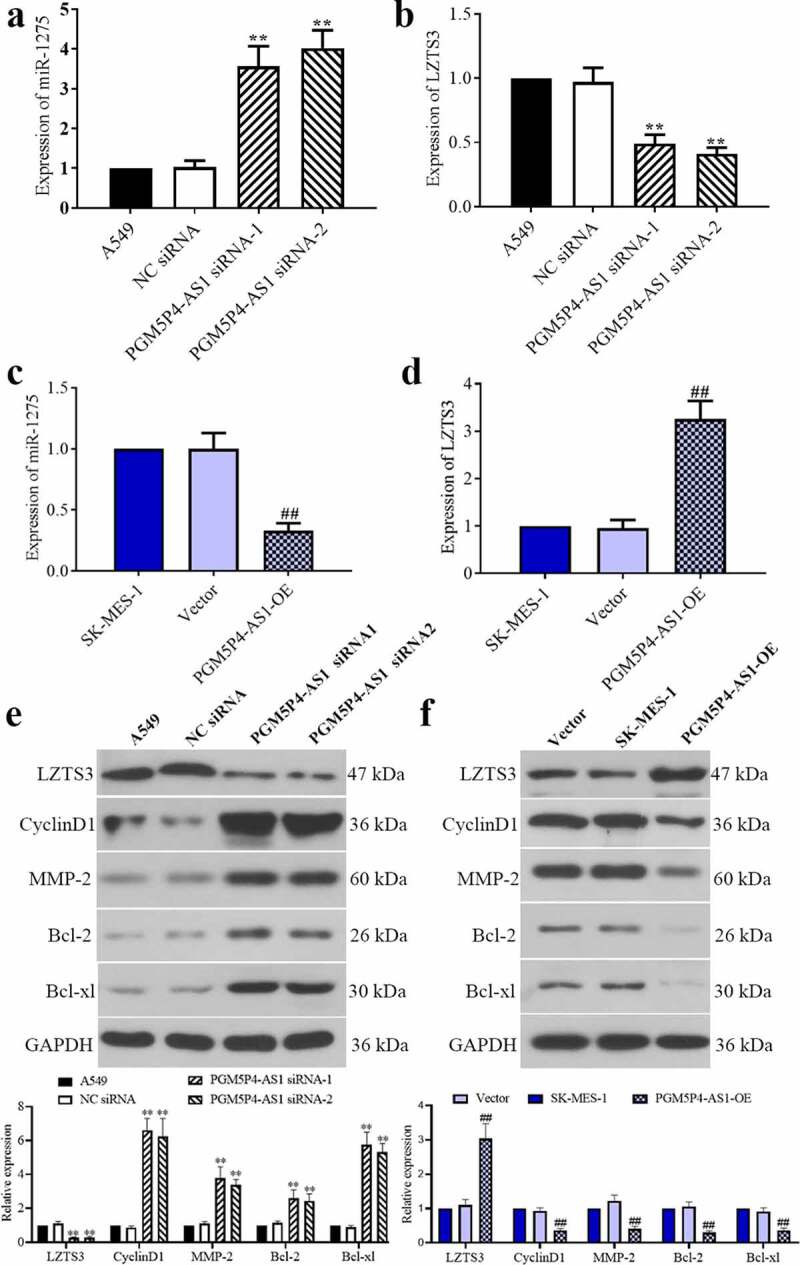
**PGM5P4-AS1 decreased the miR-1275 level and promoted the expression of LZTS3. (**a-b), Relative expression levels of miRNA-1275 and LZTS3 in A549 cells, that were interfered in PGM5P4-AS1 expression (**p < 0.01 *vs*. NC siRNA). (c-d), Relative expression levels of miRNA-1275 and LZTS3 in SK-MES-1 cells, that were overexpressed of PGM5P4-AS1 (##p < 0.01 *vs*. vector). (e-f), Protein expression levels of LZTS3, cyclinD1, MMP-2, Bcl-2, and Bcl-xl in A549 cells, that were transfected with PGM5P4-AS1 siRNA or PGM5P4-AS1-OE, respectively (**p < 0.01 *vs*. NC siRNA, ##p < 0.01 *vs*. vector)

High expression of miR-1275 can promote the cell proliferation and migration of lung cancer cells by targeting the leucine zipper putative tumor suppressor 3 (LZTS3) [20], thus, the expression of miR-1257 and LZTS3 was measured by qPCR. As shown in), down-regulation of PGM5P4-AS1 increased the miR-1257 level and reduced the LZTS3 level (3.56±0.51, 4.02±0.45 *vs*. 1.03±0.16; 0.49±0.07, 0.41±0.05 *vs*. 0.97±0.11), on the contrary, the up-regulation of PGM5P4-AS1 decreased the miR-1257 level and increased the LZTS3 level (0.33±0.06 *vs*. 1.00±0.13; 3.26±0.38 *vs*. 0.96±0.17). Western blotting was carried out to evaluate the expression levels of LZTS3 and its downstream proteins Cyclin D1, matrix metalloproteinase-2 (MMP-2), Bcl-2, and Bcl-xl. As expected, the change of LZTS3 protein level was the same as its mRNA). Further, as revealed in Figure 5(e), the down-regulation of PGM5P4-AS1 resulted in increased protein levels of CyclinD1 (6.61±0.69, 6.25±1.05 *vs*. 0.87±0.09), MMP-2 (3.79±0.66, 3.38±0.33 *vs*. 1.11±0.11), Bcl-2 (2.61±0.48, 2.45±0.40 *vs*. 1.14±0.11), and Bcl-xl (5.77±0.73, 5.32±0.51 *vs*. 0.89±0.10). On the other hand, the over-expression of PGM5P4-AS1 significantly inhibited the expression of CyclinD1 (0.37±0.05 *vs*. 0.93±0.09), MMP-2 (0.41±0.07 *vs*. 1.22±0.17), Bcl-2 (0.30±0.05 *vs*. 1.05±0.14), and Bcl-xl (0.37±0.06 *vs*. 0.91±0.11) in lung cancer cells (Figure 5(f)). These results indicated that PGM5P4-AS1 may promote the expression of the anti-cancer protein LZTS3 by the negative regulation of miR-1275.

### PGM5P4-AS1 suppressed the malignant behavior of lung cancer cells by sponging miR-1275

**Figure 6.**
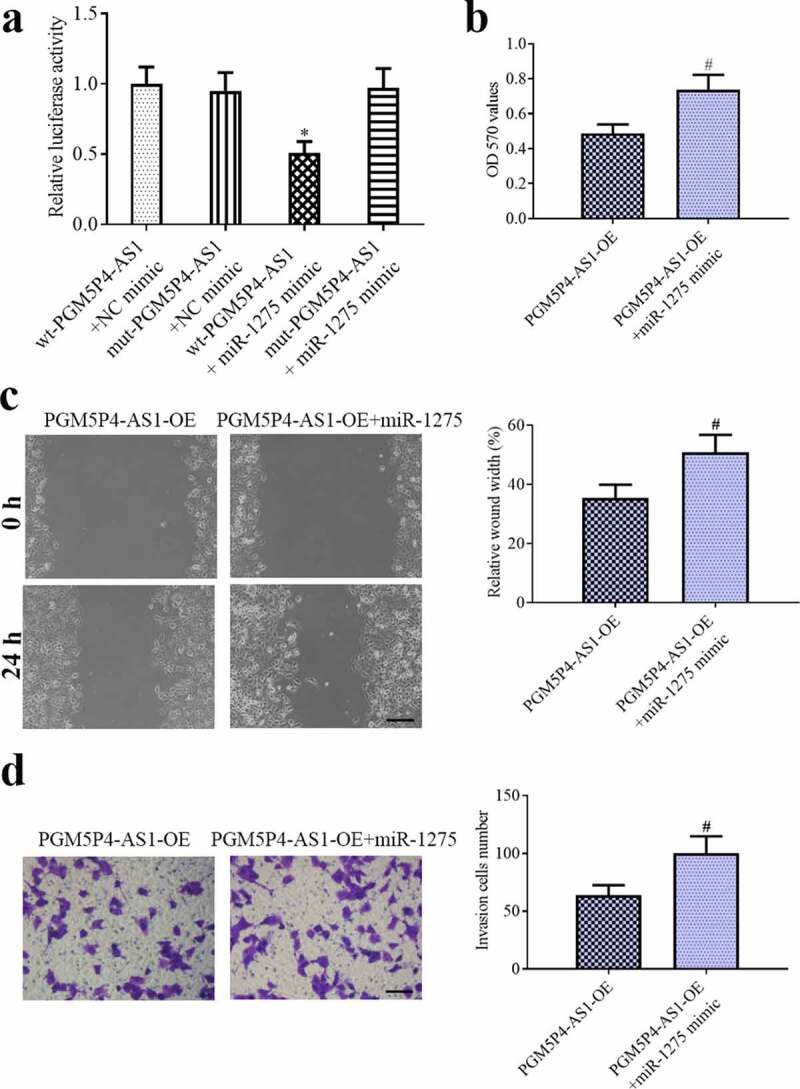
**PGM5P4-AS1 suppressed the malignant behavior of lung cancer cells by sponging miR-1275. (**a), The Dual-luciferase reporter gene showed that PGM5P4-AS1 sponged miR-1275 (*p < 0.05 *vs*. wt-PGM5P4-AS1+ NC mimic). (b), MTT assay was performed to evaluate the ability of cell proliferation (#p < 0.05 *vs*. PGM5P4-AS1-OE). (c), the migration ability of cells was assessed by wound healing assay (×100, Scale bar represented 200 μm; #p < 0.05 *vs*. PGM5P4-AS1-OE). (d), Transwell assay was used to estimate the cell invasion ability (×200, Scale bar represented 100 μm; #p < 0.05 *vs*. PGM5P4-AS1-OE)

To investigate the association between miR-1275 and PGM5P4-AS1, luciferase reporter assay was applied as described above, and the results demonstrated that PGM5P4-AS1 had the specific bind site of miR-1275 (relative luciferase activity: 0.51±0.08 *vs*. 1.00±0.12; 0.97±0.14 *vs*. 0.95±0.13; Figure 6(a)). Further, the MTT, wound healing and transwell results suggested that PGM5P4-AS1 over-expression induced suppression of the proliferation (OD 570 values: 0.74±0.09 *vs*. 0.49±0.05), migration (relative wound width: 50.79%±6.01% *vs*. 35.40%±4.52%), and invasion activities (the invasion cell number: 100.20±14.56 *vs*. 63.8 ± 8.81) of lung cancer cells were all reversed by miR-1275 mimic). These results suggested that PGM5P4-AS1 affected the malignant behavior of lung cancer cells partly by the regulation of miR-1275.

## Discussion

LncRNAs acts as a pivotal part in genome activities, thereby regulating cellular processes, even the occurrence, and development of malignant cancers. Several papers have delineated that many lncRNAs have a function in the process of lung cancer. Loewen et al. [11] reported that lncRNA HOTAIR was associated with aggressiveness and poor prognosis in lung cancer. LncRNA GAS5 could affect drug resistance in NSCLC [21]. LncRNA H19 could suppress the expression of miR-138 and miR-200a and was related to disease-free survival (DFS) time [22,23]. Ji et al. [24] demonstrated that LncRNA MALAT1 was identified as a prognostic biomarker and was associated with the survival rate in lung cancer. This study concentrated on the up-expressed PGM5P4-AS1 regulated the cellular processes and the growth of tumors by sponging miR-1275 to up-regulate the expression of LZTS3 in lung cancer.

PGM5P4-AS1, a newly discovered lncRNA, is predicted as a potential biomarker in various cancers. Most studies on PGM5P4-AS1 are performed by bioinformatics analysis and are rarely verified by experiments [17–19]. Herein, we found that PGM5P4-AS1 expression was lower in lung cancer tissues than adjacent tissues and we firstly identified its anti-tumor activity *in vivo* and *in vitro*. Accumulated evidence show that lncRNAs can interact with miRNA and further regulate its target genes at the post transcription level, which are known as ceRNAs [25,26]. Yang et al. [27] reported that lncRNA LINC01133 inhibited gastric cancer progression via sponging miR-106a-3p to regulate the adenomatous polyposis coli gene expression. Zhao et al. [28] displayed that the knockdown of lncRNA HOTAIR suppressed cell proliferation, metastasis, and apoptosis by affecting the miR-20a-5p/HMGA2 axis. These lncRNA-related ceRNAs are associated with the anti-cancer progression. Thus, we speculated that PGM5P4-AS1 might also affect the malignant phenotypes of lung cancer by the regulation of miRNA and its targets.

Recently, the dysregulation of miR-1275 was proven to affect the development and progression of various cancers. Zheng et al. [29] reported that miR-1275 facilitated the tumorigenesis and metastasis in nasopharyngeal carcinoma by up-regulating integrin β3. Xie et al. [30] found that miR-1275 was associated with radiosensitization in esophageal mediating by Wnt/β-catenin signaling pathway. Ma et al. [31]reported that miRNA-1275 promoted lung cancer cell proliferation and migration via the regulation of FOXK1. Moreover, miR-1275 was proved to be highly expressed in NSCLC and promoted the cellular processes including cell proliferation, migration, and invasion by targeting LZTS3 [20]. In the current study, the results of luciferase reporter assay showed that PGM5P4-AS1 sponged miR-1275. We found that PGM5P4-AS1 overexpression induced blocking of proliferation, migration, and invasion abilities of lung cancer cells were all reversed by miR-1275 mimic. Subsequently, scholars revealed that LZTS3 regulated the process of cell growth and was thought to be a potential tumor suppressor [32]. Liu et al. [33] demonstrated that LZTS3 was predicted to be a potential prognostic biomarker in colorectal cancer by bioinformatics analysis. In the current study, LZTS3 acted as a target of miR-1275 in lung cancer cells. LTZS3 inhibited cell proliferation by down-regulating the cell cycle pathway protein cyclin D1 and suppressed cell migration by down-regulating metastasis-related protein MMP2.

## Conclusions

Our work manifested that PGM5P4-AS1 was low expressed in lung cancer tissues. PGM5P4-AS1 could inhibit lung cancer progression by the up-regulation of the LZTS3 expression via sponging miR-1275. PGM5P4-AS1 may be a potential precise diagnosis and therapeutic target biomarker in lung cancer.

## Supplementary Material

Supplemental Material

## Highlights:

1. PGM5P4 AS1 was down-regulated in lung cancer tissues.

2. PGM5P4 AS1 inhibited lung cancer cell proliferation, migration, and invasion.

3. PGM5P4-AS1 suppressed the growth of lung cancer tumors in vivo.

4. PGM5P4-AS1 acted as a sponge of miR-1275 in lung cancer cells.

5. LZTS3 acted as a target of miR-1275 in lung cancer cells.
