# Supplementary material for: Long non-coding RNA (lncRNA) PGM5P4-AS1 inhibits lung cancer progression by up-regulating leucine zipper tumor suppressor (LZTS3) through sponging microRNA miR-1275
Source: Bioengineered. 2020 Dec 31;12(1):196–207. doi: 10.1080/21655979.2020.1860492 (PMC8806334; doi:10.1080/21655979.2020.1860492)
Supplement: Supplemental Material [file KBIE_A_1860492_SM3228.docx]

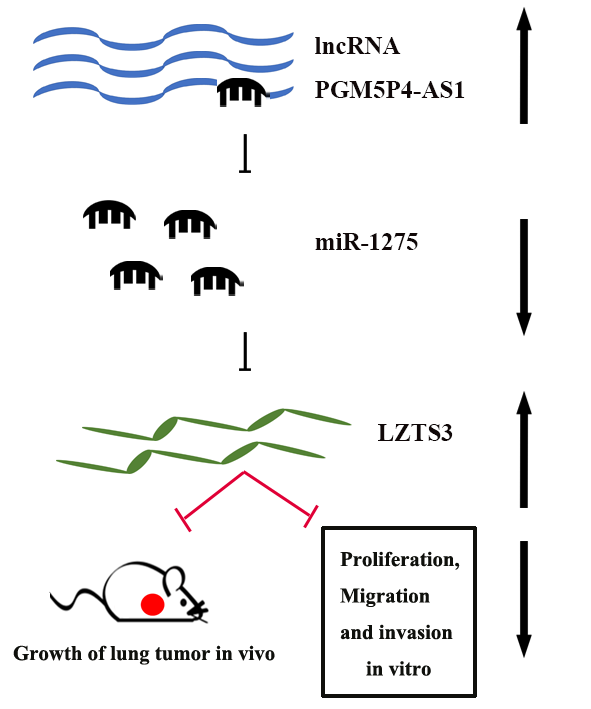


PGM5P4-AS1 inhibited the growth of tumors in nude mice and suppressed the abilities of cell proliferation, migration as well as invasion of lung cancer cells. In mechanism, PGM5P4-AS1 performed antitumor function by up-regulating the expression of LZTS3 via sponging miR-1275.
